# Mating status-dependent “choice” in competitive and noncompetitive arenas

**DOI:** 10.1093/beheco/araf080

**Published:** 2025-08-28

**Authors:** Robert J Dugand, Rowan A Lymbery, Nirjana Dewan, W Jason Kennington, Joseph L Tomkins

**Affiliations:** Centre for Evolutionary Biology, School of Biological Sciences, The University of Western Australia, Crawley, WA, 6009, Australia; Centre for Evolutionary Biology, School of Biological Sciences, The University of Western Australia, Crawley, WA, 6009, Australia; Department of Biodiversity, Conservation and Attractions, Kensington, WA, 6151, Australia; Centre for Evolutionary Biology, School of Biological Sciences, The University of Western Australia, Crawley, WA, 6009, Australia; Centre for Evolutionary Biology, School of Biological Sciences, The University of Western Australia, Crawley, WA, 6009, Australia; Centre for Evolutionary Biology, School of Biological Sciences, The University of Western Australia, Crawley, WA, 6009, Australia

**Keywords:** female mate choice, female mating status, male-male competition, sexual selection

## Abstract

To maximize their reproductive fitness, females of many polyandrous species should display mating status-dependent choice, where they mate relatively indiscriminately once to ensure reproductive output, and then become choosy and mate preferentially with higher-quality males. Despite this potential contrast in choosiness, most mate choice experiments use virgin females. Here, using a panel of 20 isofemale strains that originated from wild-caught flies, we allowed virgin and non-virgin *Drosophila melanogaster* females to choose among males from the same panel of strains. We used single-male latency trials and a series of male competition trials to help disentangle female “choices” from male-male competitive effects. Most virgin females mated within 2 h of males being introduced, compared with fewer than half of non-virgin females mating over the same period. However, despite mating more rapidly, virgin females did not mate indiscriminately, and their “choices” strongly aligned with those of previously mated females across both the single-male latency and male-male competition trials. Our results challenge the idea that virgin females mate relatively indiscriminately and show that female choice may be more stable than is generally appreciated.

## Teaser

Females of many taxa choose to mate multiples times, despite potential harmful effects of remating. Consequently, females should make more discerning mating decisions if and/or when they choose to remate. Here, we show that female vinegar flies make very similar mate choice decisions in their first and second mating, challenging theory that suggests virgin females are less choosy.

## Introduction

Owing to greater gamete and reproductive investment (Bateman 1948; Trivers 1972), females of many animal taxa are under selection to make adaptive mating decisions. Mating multiply (polyandry) and inciting sperm competition (Parker 1970) and cryptic female choice (Eberhard 1996) may allow females to circumvent the need to make active adaptive choices whilst receiving genetic benefits (Jennions and Petrie 2000). However, mating itself can be costly (Chapman et al. 1995; Kemp 2012), and fertilization is often biased towards the first (Rodrigues et al. 2020) or last (Snook and Hosken 2004) male to mate (ie dependent on mating order and not sperm quality), limiting the potential benefits of polyandry that could arise through sperm competition and/or cryptic female choice. If females exercise mate choice, they can gain direct benefits and indirect genetic benefits (Prokop et al. 2012) from their choices, but excessively choosy females run the risk of virgin death with no reproductive output (Kokko and Mappes 2005). A simple hypothesis, therefore, is that females of polyandrous species should display mating status-dependent mate choice—ie mate relatively indiscriminately once, then become choosy and mate preferentially with males that maximize their (and their offspring’s) reproductive fitness (“trade up”) (Jennions and Petrie 2000). In a recent meta-analysis, Richardson and Zuk (2022) compared the mating decisions of virgin and non-virgin females across a range of taxa. They found no evidence for an effect of mating history on three key traits (reproductive isolation, inbreeding avoidance, and sexually transmitted disease) (Richardson and Zuk 2022). However, the authors noted that empirical studies of female mate choice were dominated by the use of virgin females (Tanner et al. 2019; Richardson and Zuk 2022), despite theory suggesting that “intersexual selection…is mainly the domain of mated females” (quote from Kohlmeier et al. (2021)). Therefore, any mating status-dependent effects (or lack thereof) remain to be resolved via studies that incorporate direct comparisons of mate choices in virgin and non-virgin females.

A large body of the theoretical and empirical research on female mate choice is centered on the paradox of the lek, where persistent female choice for indirect genetic benefits should erode genetic variation and preclude the very benefits on which choice was based (Kirkpatrick and Ryan 1991). Among the proposed resolutions to the lek paradox (Pomiankowski and Møller 1995; Kotiaho et al. 2008), genic capture (Rowe and Houle 1996; Tomkins et al. 2004), where sexually selected trait expression depends on genome-wide mutation load, has burgeoning support (Kotiaho et al. 2001; Lumley et al. 2015), with genetic variation maintained through the constant influx and transient persistence of deleterious mutations that occur across the entire genome (Dugand et al. 2019; Parrett et al. 2022). In this case, choosy females receive “good genes” benefits, producing offspring with a smaller burden of deleterious recessive mutations.

Often alongside directional female preferences (Colegrave et al. 2002), there are also well documented effects of genetic compatibility (Tregenza and Wedell 2000; Hoffman et al. 2007). Choosy females mate with males that maximize their offspring’s fitness, but there is no one best male genotype—the best male genotype depends on the genotype of the female. For example, owing to deleterious effects of inbreeding, females may choose to mate with unrelated males (Hoffman et al. 2007). Here, genetic variation is maintained because the benefits of choice are nonadditive (Kotiaho et al. 2008).

Importantly, the lek paradox depends on female choice, yet female mating biases can arise through various means. For example, if females passively mate with competitively superior males, then the paradox is no more (Kotiaho and Puurtinen 2007). Indeed, even in lek mating systems—the archetypal example of female mate choice—male-male competition contributes substantially to variance in male reproductive success (DuVal and Kempenaers 2008). The relative importance of male-male competition *versus* female choice in determining mating biases is important for sexual selection theory, yet studies tend to focus on one mechanism or the other (Kotiaho and Puurtinen 2007; Hunt et al. 2009).

*Drosophila melanogaster*, a species which is polyandrous and displays last-male sperm precedence (Laturney et al. 2018), is an ideal system for testing questions about mating status-dependent choice, genetic benefits, and the impact of male-male competition thereon. In *D. melanogaster*, virgin females mate rapidly, whereas non-virgin females have a much lower propensity to mate. This is often interpreted to imply that virgin females mate relatively indiscriminately and non-virgin females are much choosier. However, male *D. melanogaster* deliver courtship songs that activate sensory pathways in females that lead to vaginal opening and, subsequently, mating (Wang et al. 2021). Upon mating, males transfer seminal fluid proteins that inhibit these sensory pathways and temporarily preclude remating (Wang et al. 2021). Hence, the mechanisms underlying reduced remating rates do not necessarily suggest that non-virgin females make more discerning remating decisions; rather, they are mechanically prohibited from remating for some time. Moreover, genetic benefits of competitive mate choice have been demonstrated through artificial selection experiments using both virgin (Promislow et al. 1998) and non-virgin (Dugand et al. 2018) females, suggesting that mating biases may be similar regardless of the mating status of the female.

Despite the mechanistic arguments presented above, mating status-dependent choice has been demonstrated at least twice in *D. melanogaster*. First, Kohlmeier et al. (2021) showed that when females from one laboratory population were presented with males from two, independent laboratory populations, non-virgin females displayed a strong preference, but virgin females did not. Second, Chechi et al. (2022) evolved populations under male- and female-biased mating regimes. Following experimental evolution, virgin and non-virgin females preferred males from the male-biased regime, but this preference was significantly stronger among non-virgin females. In both studies, females chose among competing males from contrasting populations or selection regimens, and, therefore, the extent and consequences of mating status-dependent choice within a population remains unclear. Furthermore, how male-male competition and female choice might affect mating status-dependent mating biases in *D. melanogaster* is unclear. There is extensive evidence for sexual conflict in this species (Chippindale et al. 2001), and larger males are more harmful to females than smaller males (Pitnick and García–González 2002; Friberg and Arnqvist 2003), with some evidence suggesting that larger males may be more strongly favored when male-male competition is present (De Nardo et al. 2021). Hence, the costs and benefits of mating status-dependent choice might differ in the presence *versus* absence of male-male competition, as they do in *D. simulans* (Taylor et al. 2008), a sister species of *D. melanogaster*.

Here, we tested mating status-dependent choice using a panel of 20 isofemale strains of *D. melanogaster*. In five replicate blocks, each with four different isofemale strains, we measured the mating success of males from all isofemale strains in competitive and noncompetitive arenas when presented to virgin and non-virgin females from all four isofemale strains. Notably, virgin and non-virgin females differ in their ‘responsiveness’ to mating—virgin females have a much higher propensity to mate (ie they are more responsive)—but this does not preclude them from being *choosy*. ‘Choosiness’, the focus of this study, refers to the propensity of females to mate with certain males over others (Ratterman et al. 2014; Reinhold and Schielzeth 2015). Variation among isofemale strains in the mating success of males is, therefore, informative about female choosiness. Comparing this variation across contexts (eg virgin *versus* non-virgin females) provides insight into the presence of mating status-dependent choice, and the impact of male-male competition thereon. As noted above, contrasting choices of virgin and non-virgin females in different contexts may result in different costs and benefits. To test whether female choices, or male-male competitive effects, would result in genetic benefits or costs, we also quantified various measures of fitness. Overall, our results suggested that virgin females were not only choosy, but also displayed very similar mate choices to non-virgin females within the two social contexts.

## Methods

### Fly collection and generation of isofemale strains

Individuals of *Drosophila melanogaster* were collected from several banana bunches across a banana plantation in Mareeba, Queensland, Australia in December 2021. Non-virgin females were placed singly into 50 mL glass vials with 10 mL sugar-maize-yeast media to lay eggs. From each female, we harvested a single son or daughter. We then crossed lines by mating a male from one line to a female from a different line. These crosses produced a total of 60 isofemale strains, each with four distinct sets of chromosomes (David et al. 2005). Isofemale strains were each maintained across five glass vials, mixed each generation, at a census size of approximately 100 flies (~10 males and ~10 females per vial) for 5 to 10 generations. Twenty isofemale strains were the focus of this study. We note that there would inevitably be some inbreeding within isofemale lines prior to the start of the experiment. However, the method of line maintenance (dividing and mixing flies within each line each generation) would substantially reduce such effects.

For panels of isofemale strains, among-strain variation largely reflects genetic variation, and within-strain variation reflects both segregating genetic variation and environmental variation (David et al. 2005). Use of isofemale strains in this study—where we can produce many males for each of 20 independent genetic lineages—ensures that we can test the replicated choices of virgin and non-virgin females across contexts. Moreover, testing the choices of females from different genetic backgrounds enables us to link their choices with the potential additive (eg do females from different genetic lineages all prefer similar males, *sensu* good genes-type benefits) and nonadditive (eg do females from different genetic lineages prefer different males, *sensu* compatibility-type benefits) genetic benefits they receive. Crucially, the isofemale strain approach is a particularly powerful tool for testing genetic correlations between traits (David et al. 2005), and it is the genetic correlation between virgin and non-virgin female choices that is the main focus of this study.

### Experimental flies

**Fig. 1.**
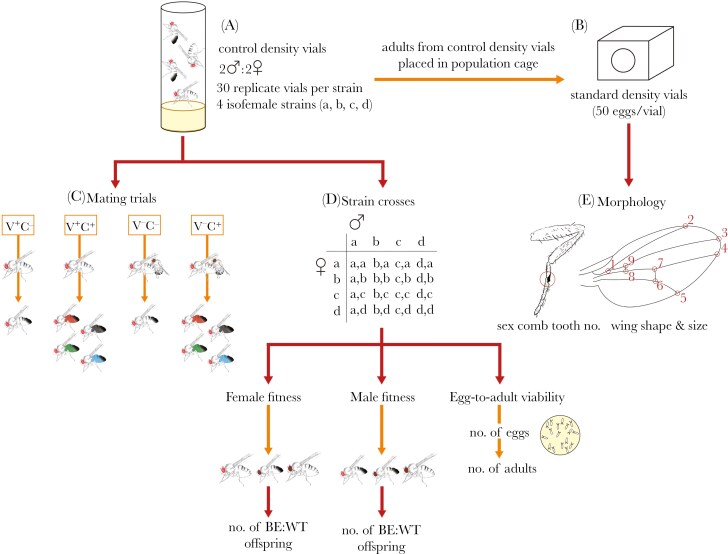
Schematic of the experimental design. From each of four isofemale strains (a, b, c, and d) of *Drosophila melanogaster*, flies were collected and placed into vials, each with two males and two females (**A**; controlled density vials). After 4 d, these flies were transferred to a single population cage (one cage per strain) to lay eggs (**B**). Eggs were collected and placed into vials at a density of 50 per vial (standard density vials). Most flies emerging from the controlled density vials were used in mating trials (**C**). We performed competitive (C^+^) and noncompetitive mating trials (C^-^), where females were either virgin (V^+^) or non-virgin (V^-^). Non-virgin females were first mated to males from an isogenic, brown-eyed strain. In competitive trials, one male from each strain was present with their abdomens dyed different colors, and females from all four strains were presented (singly) to these groups of males. All noncompetitive trials had one male and one female, with males from all four isofemale strains presented to females from all four isofemale strains. The remining flies emerging from the controlled density vials were crossed within and between strains in a full factorial design (**D**; strain crosses). For fitness, these crosses involved three replicate vials of two males and three females, and the offspring emerging from these crosses were used to measure male and female fitness, where they were competed against a brown-eyed fly of the same sex for access to a brown-eyed fly of the opposite sex. Fitness was measured (the log ratio of the numbers of wild-type [WT] and brown-eyed [BE] flies produced) 10 times for each sex and cross combination. For egg-to-adult viability, each cross combination was replicated 10 times, each with one male and one female, and the numbers of eggs and adults they produced after being held together for 22 h were recorded. Finally, flies emerging from standard density vials were used to measure wing shape and size and sex comb tooth number (**E**). In this schematic, smaller flies with dark abdomens are male, larger flies with striped abdomens are female, and flies with dark-brown eyes are from an isogenic brown-eyed strain. This design was replicated five times, with four different isofemale strains represented in each randomized block.

The following experimental design was replicated across five blocks, each with four isofemale strains (see Fig. 1 for a schematic of the experimental design).

From each of four isofemale strains (haphazardly chosen from the 60-strain panel), we collected flies and placed two females and two males into each of 30 replicate, ‘controlled density’ vials (ie 120 vials total, 30 vials per strain; Fig. 1A) and left them to mate and lay eggs over 4 d, ensuring larval density was not too high. Offspring that emerged from the controlled density vials were used in experimental mating trials (Fig. 1C) and to measure egg-to-adult viability and fitness (Fig. 1D).

After the initial 4 d of mating and egg laying, the two male and female flies per vial were transferred (Fig. 1A to Fig. 1B, orange arrow) to a combined population cage for their respective isofemale strain (ie the 60 males and 60 females from an isofemale strain combined in a cage). The four population cages (one per isofemale strain) each contained a Petri dish with grape juice set with agar and a smear of Baker’s yeast paste, onto which females lay eggs (Fig. 1B). From these dishes, we collected eggs and placed them into vials at 50 eggs per vial. Offspring emerging from these vials were used to measure wing shape and size and sex comb tooth number (Fig. 1E), morphological traits that have been linked to male mating success (Partridge and Farquhar 1983; Partridge et al. 1987; Ng and Kopp 2008; Dugand et al. 2018).

### Isogenic, brown-eyed flies and the making of non-virgin females

Our goal was to measure the mate choices of virgin and non-virgin females; hence, some females first needed to be mated before assessing their choices (see below). We were not interested in how the first mating directly impacted the second mating (ie the phenotypic correlation); for example, there is genetic variation in male-induced refractory periods for female remating (eg (Service and Vossbrink 1996)). Rather, our focus was on the genetic (co)variation in the mating decisions of females across contexts (eg virgin *versus* non-virgin). Therefore, to generate non-virgin females for our trials, we opted to first mate females to males that came from a single isogenic strain, thus minimizing variation in female remating rates due to first male effects.

The isogenic strain used in this study carried a brown-eyed recessive mutation. Note that we use this population of flies twice. First, we use only the brown-eyed males to mate with females that are subsequent used in non-virgin choice trials (ie the brown eye color is irrelevant). Second, we measure competitive fitness, where eye color is used to compare the numbers of brown-eyed and red-eyed (wild-type) offspring (further details below).

To collect flies from the brown-eyed population, we placed two males and three females into each of 60 replicate vials for 4 d, and collected offspring that emerged from these vials. Emerging flies were collected as virgins, with males used for mate choice trials, and males and females used to measure competitive fitness.

### Mating trials

To determine the choosiness of females, and how this varied across contexts (virgin *versus* non-virgin females, with and without male-male competition), we performed a series of mate choice trials. Competitive and noncompetitive trials were performed separately, on flies reared at different generations. Within each of these trial types, virgin and non-virgin females from all four strains were assayed simultaneously. For all trials, observers were blind to the isofemale strain and mating status information.

Notably, in this experiment, flies from different isofemale strains never developed in the same vial; however, within isofemale strains, a male-female pair in a given mating trial had an approximate 1 in 30 chance of being from the same rearing vial. Whether matings are more or less likely among familiar and/or related flies is somewhat unclear (Ödeen and Moray 2008; Tan et al. 2012; Lizé et al. 2014). Regardless, any effect should be negligible (due to the low probability of two flies being drawn from the same vial) and rare occurrences would be randomly distributed among the isofemale strains.

#### Noncompetitive mating trials.

We collected flies from the controlled density vials within 8 h of emergence, before they reached sexual maturity. These flies were maintained in unisex groups of 15 to 20 individuals, with live Baker’s yeast added to female food media. At 2 to 3 d old, half of the females were mated to brown-eyed males (Fig. 1C, V^-^C^-^) in groups of 30 to 40 flies at an approximate 1:1 sex ratio. Brown-eyed males were discarded the following day, allowing females to mate, but largely precluding remating (eg (Best et al. 2012)). We assume most females would have mated during this time (evidenced by the vastly lower mating probabilities in the non-virgin trials, see **Results**), and any unmated females would be randomly distributed across subsequent mating trials. All females were then housed in vials (15 to 20 flies per vial) containing food media supplemented with live Baker’s yeast, and were transferred to fresh vials after 4 d. We performed noncompetitive mating trials 2 to 3 d after being transferred to fresh vials, ie 6 to 7 d after first mating, when flies were approximately 8 to 10 d old. Females are relatively unresponsive to mating in the first few days after mating, and their productivity plateaus after ~10 to 14 d post mating (eg (Stewart et al. 2007)), at which point they should theoretically be less choosy. Hence, the 6 to 7 d inter-mating interval reflects a trade-off, where we get a reasonable sample size from relatively choosy females. Note that virgin and non-virgin females were the same age when used in mate choice trials.

All mating trials were conducted in 50 mL glass vials with 10 mL food media. Males from all four isofemale strains were presented to females from all four isofemale strains and two mating statuses (16 combinations per mating status per block). To do so, we first placed virgin males singly into numbered vials. We then added one female (from a given strain and mating status) to each vial and recorded the time to mating of each pair. To ensure approximately equal representation of the 32 experimental groups among all trials in a block, we used a semi-stratified design for conducting the trials. For example, for 20 consecutive trials (here, referred to as a ‘group’), we used females from a single strain and mating status, within which there would be five males from each strain. Likewise, 80 consecutive trials would feature virgin females (four groups, including females from all strains), followed by 80 non-virgin trials. Nevertheless, each group of trials was performed multiple times, and there was substantial temporal overlap among all groups. Additionally, because, vials were labeled with a number only, observers checking for matings and recording mating latency (these roles were performed by volunteers) were blind to the mating status of the female and the strain ID of both the male and female, removing any potential observer bias. The trials were suspended after 2 h, at which point most virgin females had mated, and activity tends to decrease (*D. melanogaster* are most sexually active at “dawn” and “dusk,” with activity decreasing away from these times). On average, 19 virgin, and 15 non-virgin, trials were performed for each of the 16 strain combinations per block. Across the five blocks, we performed 1,190 non-virgin mating trials and 1,539 virgin mating trials.

#### Competitive mating trials.

Flies used in competitive trials were reared identically to those used in the noncompetitive trials.

To distinguish males from different strains during competitive mating trials, we transferred male flies into vials containing live yeast impregnated with food dye 1 to 4 d before the competitive mating trials. The duration that males were fed dyed yeast varied between blocks, but not between isofemale strains within a block. Males from different strains were fed different colored yeast which, once consumed, is visible in the abdomen (see Fig. 1C, C^+^). Although males from a given isofemale strain were only one color, each color was represented in each of the five blocks, allowing us to model the overall effect of color as a categorical fixed effect. Overall, color had minimal effect on competitive mating success (also see Verspoor et al. 2015), with the percentage of matings across the five blocks all being close to 25%: 27.76% (black), 23.77% (blue), 21.17% (green), and 27.30% (red).

Competitive mating trials were conducted in numbered vials, with one male from each isofemale strain (four males total) being presented to a single virgin or non-virgin female from one of the four isofemale strains. Vials were scanned, mating pairs removed by aspiration, the time-to-mating recorded, and males frozen and scored for color. Where necessary, the isofemale strain ID of the successful male was confirmed by assessing the colors of the three unsuccessful males. Color scoring was performed blind to the mating status and strain ID of the female. Mating trials were suspended after 2 h. On average, 21 virgin females, and 32 non-virgin females per isofemale strain were used in competitive trials. Across the five blocks, 715 females mated; however, on 70 occasions, the mating pair separated before being identified, flies escaped, or we could not identify the color of the successful male. We discarded all data where a successful male could not be confidently identified.

### Measuring potential costs and benefits of mate choice

#### Morphology.

Size strongly influences male mating success in *D. melanogaster*, with successful males being larger in most (eg (Partridge and Farquhar 1983; Partridge et al. 1987; Dugand et al. 2018)), but not all (eg (Robinson et al. 2012a; De Nardo et al. 2021)), contexts. Determining how size influences mating success across the four contexts (virgin *versus* non-virgin females in competitive and noncompetitive contexts) may be invaluable for understanding adaptive choices. For example, do non-virgin females mate less frequently with larger, more harmful (Pitnick and García–González 2002) males? Additionally, wing shape (Menezes et al. 2013) and sex combs (Ng and Kopp 2008) have also been shown to affect male mating success. Thus, we measured these three morphological traits to determine their role in mate choice across the four contexts. Our measure of size was wing length, which is highly correlated with body size (Robertson and Reeve 1952).

To measure wing shape and length, we removed the right wing of male flies and mounted them on double-sided tape. We then photographed the wings and located nine landmarks (Fig. 1E) using *tpsdig2* (Rohlf 2015). Wings were then aligned using full Procrustes fit in *morphoJ* (Klingenberg 2011). Fourteen principal components (PCs) of the size-corrected XY coordinates have variance; we used these 14 traits plus wing size to remove six wings that were multivariate outliers based on Mahalonobis distance (χ^2^_crit_ = 37.70, d.f. = 15, α = 0.001). Wing length was scored as the distance, in mm, between the third and ninth landmarks (Fig. 1E). Our measure of wing shape was PC1 (capturing 17.6% of the shape variation), which describes an axis of wing shape variation where the second and fifth landmarks are more proximal and distant (*versus* distal and closer together; rounder *versus* narrower wing) with a rounder (*versus* pointier) wing tip (see Fig. 1E for wing landmarks, and Table S3 for eigenvector loadings for PC1).

Finally, for all males from which we measured wings, we simultaneously removed their front legs, mounted them on double-sided tape, and summed the number of teeth in the two sex combs (Fig. 1E).

On average, we measured 39 males (that emerged across six vials) per strain (780 males in total).

#### Fitness and egg-to-adult viability.

To determine whether female mating biases would result in indirect genetic benefits (eg good and/or compatible genes), we measured the fitness and egg-to-adult viability of flies that were the products of all isofemale strain crosses (Fig. 1D). That is, within a block, virgin flies emerging from each of the four isofemale strains were crossed within and between isofemale strains in a full-factorial design (ie 4 × 4 = 16 crosses in total).

To measure fitness, each of the 16 crosses were replicated three times, and each vial contained three females and two males. These small groups were housed together in vials for 4 d before being discarded. From each of the 12 between-strain crosses, we collected up to 10 virgin sons and daughters and scored them for their fitness. For each of the four within-strain crosses, we had up to 20 replicates; we had twice the number of within-strain crosses because all between-strain crosses are reciprocal (10 with males from X and females from Y, and *vice versa*). To measure fitness, a single virgin wild-type male or female was placed into a vial with one virgin brown-eyed male and one virgin brown-eyed female (Fig. 1D, female and male fitness). All trios were held together for 6 d before being discarded. After 2 wk, emerging offspring were frozen and photographed. Eye color was scored from these photographs by one observer that was blind to the strain IDs. A total of 120,361 flies were scored for eye color across 1,478 photos.

Fitness was scored as (Reddiex et al. 2013):


$$\begin{array}{l} w=ln\left(\frac{wt+1}{be+1}\right) \end{array}$$

where wt is the number of wild-type offspring and be is the number of brown-eyed offspring.

Only flies that survive development are exposed to fitness trials, and these may be a biased representation of the quality of sons and daughters from a given cross combination (Polak and Tomkins 2013). For example, if low-fitness flies died before maturing, and such individuals were more common for within-strain crosses, then this developmental selection would cause us to overestimate the fitness of within-strain crossed flies. Therefore, we also measured the egg-to-adult viability of all cross combinations (independent of the fitness assay). To measure egg-to-adult viability, we set up single-pair crosses (ie one male and one female in a vial) in the full-factorial design outlined above (Fig. 1D, strain crosses), and held pairs together for 22 h to mate and lay eggs. After 22 h, flies were discarded, and the eggs counted. Fourteen days later, vials were inverted and frozen and the number of adults counted. Each cross was replicated up to 32 times, though most within-isofemale strain crosses were replicated 20 times, and between-isofemale strain crosses 10 times. A total of 15,235 eggs and 10,100 adults were counted across 1,155 vials. We scored egg-to-adult viability using the fitness equation presented above, but substituting wt with the number of adults, and be with the number of eggs that failed to reach the imago stage.

### Statistical analysis

Statistical analyses were all performed within the Innocent and Trusting R environment (R Core Team 2022). Bayesian mixed models were run with *brms* (Bürkner 2017), and maximum likelihood mixed models with *lme4* (Bates et al. 2015). We estimated marginal means using the *emmeans* package (Lenth 2023). Figures were produced using *ggplot2* (Wickham 2016) and *GGally* (Schloerke et al. 2021).

Our aim was to compare how choosy females were across contexts. Here, in any given mating trial, the isofemale strain from which the female was derived (henceforth referred to as F_strain_), the isofemale strain from which the male was derived (M_strain_), and their interaction (among other effects), could affect the propensity to mate. Variance in F_strain_ would largely reflect differences in female responsiveness to mating across strains, and is not the focus of this study. Variance in M_strain_ largely reflects differences in male mating success across strains and, therefore, differences in female mating decisions (although see **Discussion**). Likewise, F_strain_-by-M_strain_ interaction variance indicates that females from different strains “choose” different males, which may reflect compatibility-type mating decisions. These latter two variance components are the focus of this study. Importantly, the relatively magnitudes of these variance components, and the covariances across contexts, are informative about mating status-dependent mate choice and the impact of male-male competition thereon.

#### Male mating success.

To evaluate mating status-dependent choice and male-male competitive effects, we generated a 4 × 4 covariance matrix for mating success in the four experimental contexts: when females were virgins and there was male-male competition (V^+^C^+^) or no male-male competition (V^+^C^-^), and when females were non-virgin and there was male-male competition (V^-^C^+^) or no male-male competition (V^-^C^-^). In the covariance matrix, the variances in M_strain_ in each of the four contexts were on the diagonals, and the covariances in M_strain_ across contexts off the diagonals.

We first ensured that the data were comparable across the four contexts. For the competitive data, males either won or lost. However, males within a vial were not independent. To ensure that the unit of replication was vial, we adopted a sampling approach at the vial level, where we randomly sampled one male per vial and assigned them as successful or unsuccessful. Mating success was thus approximately 25% in the competitive contexts (it could be slightly higher or lower than 25% depending on the random sample of males). We repeated this sampling approach with 100 different sets of males (see below).

In the noncompetitive mating trials, mating success was higher than 25%. Therefore, to match the 25% mating success of the competitive trials, in the noncompetitive trials, we used the latency to mate data to score single pairs as mated or not at the 25^th^ percentile. This was performed independently for all blocks and female mating statuses. On one occasion (block three, non-virgin), mating success was only 21%, and so the raw data were used.

Although we had a similar number of males in the competitive and noncompetitive trials, the sample size is the number of trials, and thus we had about four times more data to estimate noncompetitive mating success, where all trials were independent. Moreover, in the competitive trials, no mating results in loss of data, such that the sample size was relatively low for V^-^C^+^ trials. The effective number of trials were: 1,190 (V^-^C^-^), 1,539 (V^+^C^-^), 273 (V^-^C^+^), and 379 (V^+^C^+^).

We performed the statistical analysis within the Bayesian framework using *brms* (Bürkner 2017), setting up separate models for each context. The response variable was always binary (success or failure), and was modeled using the Bernoulli family. For the noncompetitive contexts, M_strain_ and F_strain_ were modeled as random effects (see Figure S3 for F_strain_ results). For the competitive contexts, color was included as fixed effect, and M_strain_ as a random effect. We did not include a M_strain_-by-F_strain_ random effect because preliminary tests showed this component was effectively zero (Table S1). Likewise, we did not include block as a fixed effect, as mating success was ~25% in all blocks for all contexts. We used default priors, and performed 20,000 iterations for each of four chains with a thinning interval of 10 (posterior distribution with 4,000 samples).

We ran this model 100 times where, for each model, we randomly sampled a different set of males for competitive mating success. That is, we randomly selected one male per vial (25% chance of choosing the successful male) to include in the analysis. The pooled posterior distribution of estimates accounts for the random samples of males, while having no effect on noncompetitive mating success. In essence, it is equivalent to performing 100x more iterations, and does not affect estimates or credible intervals.

In these analyses, the variances (ie the diagonals of the M_strain_ covariance matrix) are constrained to be positive. While their relative magnitudes are informative about female choosiness (low variance suggests non-choosy), we do not use credible intervals (CI) to test whether variances are >0. Critically, however, the correlations (which are directly output from *brms* analyses) are only constrained to be −1 to 1, and CI can be used for hypothesis testing. We statistically tested the 95% CI of the correlations against 0 using the *mode_hdci* function in the *tidybayes* package (Kay 2023). We used the mode and highest density credible intervals because the posterior distributions were right-skewed for some variance components

We chose to take a Bayesian approach to account for the sampling of males and to estimate correlations among all contexts in a single analysis. For the noncompetitive mating trials, we confirmed the results and interpretation using maximum likelihood models implemented in *lme4* (Bates et al. 2015), where we statistically tested variances and covariances using likelihood ratio tests (Table S1) and jack-knifing, respectively. The maximum likelihood results, which are reported in full in the Supplementary Material, supported our Bayesian analysis.

#### Morphology and male mating success.

We first confirmed that there was among-strain variance for the three morphological traits (Table S4). We used the R packages *lme4* (Bates et al. 2015) and *glmmTMB* (Brooks et al. 2017) to fit (generalized) linear mixed effects models on each morphological trait, with each model including a categorical fixed effect of block, and strain and vial (nested within strain) as random effects. Random effects were tested using likelihood ratio tests, and block effects tested using Wald type II tests implemented using the *Anova* function in the *car* package (Fox and Weisberg 2019).

To determine whether morphological traits affected male mating success, we performed multiple regression analyses. Given the lack of statistical support for M_strain_ variance in competition trials (see **Results**), we focused only on the noncompetitive data. We calculated M_strain_ means for mating success to virgin and non-virgin females using the binary data outlined above. We then calculated the isofemale strain means for the three morphological traits (wing length, wing shape, and sex comb tooth number). Using linear models implemented in R (R Core Team 2022), we then tested the effects of wing size, wing shape, and sex comb size on male mating success separately for virgin and non-virgin females.

#### Fitness, egg-to-adult viability, and indirect genetic benefits.

Of the 1,154 vials set up to measure egg-to-adult viability, 511 had no eggs, and a further 59 produced no adults. Those with no eggs are uninformative for egg-to-adult viability, and those with no adults could simply reflect no mating (virgin *D. melanogaster* females do lay some unfertilized eggs). Therefore, we removed these data prior to analysis.

We first aimed to partition the variance, and assess the inbreeding effects, on viability and fitness. Using *lme4* (Bates et al. 2015), we fit linear mixed effects models with M_strain_, F_strain_, and their interaction included as random effects, and block and cross (within- *versus* between-strain) included as categorical fixed effects. Fitting cross as a fixed effect allowed us to separate inbreeding effects (ie lower viability/fitness of within-strain crosses) from other compatibility-type effects (ie high/low viability/fitness of specific male-female isofemale strain combinations, excluding inbreeding). All three traits were modeled as Gaussian responses. To improve model fit in each analysis, we removed outliers that were >2IQR from 0, based on the residuals of each model. We removed 34, 61, and four data points for male fitness, female fitness, and egg-to-adult viability, respectively. We then fit the models a second time, excluding these outliers.

Our goal was to test whether the choices of females related to the fitness outcomes, and we aimed to explore through multivariate analyses. However, we observed contrasts in which random effects explained variance in these traits (see **Results**), precluding these subsequent analyses.

## Results

### Mating status-dependent choice and male-male competition

Virgin females had a higher propensity to mate, with 91% (V^+^C^-^) and 93% (V^+^C^+^) of virgin females mating, and only 39% (V^-^C^-^) and 47% (V^-^C^+^) of non-virgin females mating, in their respective contexts.

The posterior distributions for M_strain_ variance in competitive mating success were heavily right-skewed (Figure S1) and 2.5% credible intervals were close to zero (Table 1), suggesting that this component of variance was low and/or we did not have sufficient statistical power to detect variance. By contrast, there was support for M_strain_ variance in noncompetitive mating success for both virgin (V^+^C^-^) and non-virgin (V^-^C^-^) females (Table 1; Figure S1; see Table S1 and Supplementary Text for likelihood ratio test results from maximum likelihood analyses). Again, we stress that these results do not reflect a contrast in M_strain_ variance in the presence *versus* absence of male-male competition, but a stark difference in statistical power (see **Methods**). The mode for M_strain_ variance was slightly lower for V^+^C^-^ compared to V^-^C^-^, but credible intervals overlapped substantially (Table 1).

**Table 1.** Posterior modes and 95% highest density credible intervals (below, italicized) for the M_strain_ (ie the isofemale strain from which males were derived) parameters. Diagonals are the M_strain_ standard deviations within each context, and correlations between contexts are shown below the diagonal. Females used in mating trials were virgin (V+) or non-virgin (V-), and were presented to one (C-) or four (C+) males. Bolding indicates that the 95% CI do not overlap zero.

	V + C+	V-C+	V + C-	V-C-
V + C+	0.27			
	*0.00, 0.89*			
V-C+	0.46	0.41		
	−*0.63, 0.93*	*0.00, 1.03*		
V + C-	0.43	0.18	0.32	
	−*0.54, 0.93*	−*0.64, 0.88*	*0.16, 0.56*	
V-C-	0.28	0.02	**0.80**	0.38
	−*0.59, 0.89*	−*0.75, 0.79*	***0.17, 0.99***	*0.18, 0.66*

We found statistical support for an M_strain_ correlation across the V^+^C^-^and V^-^C^-^ contexts (Table 1; Figures S1 and S2; Supplementary Text), where the lower 2.5% credible interval for the correlation was above zero. Credible intervals for all other correlations were wide, with values of approximately −0.5 to 1.0 all compatible with our data (Table 1). We note, however, that simple correlations between mating success to virgin and non-virgin females, calculated from the strain means (ie what is often used for hypothesis testing), were 0.63 in the noncompetitive arena, and 0.74 in the competitive arena (see Table S2), suggesting a strong alignment in the choices of virgin and non-virgin females within each context.

Taken together, our results strongly suggest that virgin females are not only exerting a choice over the males with which they mate, but that they make similar mating decisions to non-virgin females, at least within a noncompetitive environment.

### Morphology and male mating success

For V^+^C^-^ mating success, we found statistical support (*F*_1,16_ = 5.87, *P* = 0.028) for an effect of wing size on male mating success, where males with larger wings had shorter mating latencies (see Table S2). There was no evidence for an effect of wing shape (*F*_1,16_ = 0.22, *P* = 0.646) or sex comb tooth number (*F*_1,16_ = 011, *P* = 0.744) on mating success. There was a similar effect of wing size on male mating success in the V^-^C^-^ context, albeit without statistical stupport (*F*_1,16_ = 3.13, *P* = 0.096), and no effects of wing shape (*F*_1,16_ = 0.97, *P* = 0.339), or sex comb tooth number (*F*_1,16_ = 0.14, *P* = 0.713).

### Viability, fitness, and indirect genetic benefits

Male fitness and egg-to-adult viability were both significantly higher for between-strain compared to within-strain crosses (Table 2). A similar trend was evident for female fitness, albeit non-significant (Table 2). There was statistical support for M_strain_-by-F_strain_ variance for both male and female fitness, and for F_strain_ variance for egg-to-adult viability (Table 2). However, there was no statistical support for M_strain_ variance in any trait (Table 2). This observation contrasted with the presence of variance observed for mating success, where there was M_strain_ variance, but no M_strain_-by-F_strain_ variance. Our goal was to test the M_strain_ (and interaction) correlation across mating success and fitness traits. However, given that there was M_strain_ variance in mating success but not fitness (and *vice versa* for the interaction variance), this precluded any meaningful test of covariance between mating success and fitness or egg-to-adult viability.

**Table 2.** Linear mixed effects model results for male and female fitness and egg-to-adult viability. For the cross fixed effect (within- *versus* between-strain cross), estimated marginal means (emm) are shown with their standard errors (se). All measures of fitness and viability were log ratios. For fitness [egg-to-adult viability], values ~0 imply comparable numbers of brown-eye [dead] and wild-type [alive] offspring; values > 0 imply more wild-type [alive] offspring (higher fitness [viability] of experimental flies); values < 0 imply more brown-eyed [dead] offspring (lower fitness [viability] of experimental flies).

Trait	Effect	Cross level	emm (se)	Variance (x100)	d.f.	χ^2^	*P*
Male fitness	Block (fixed, categorical)				4	154.42	**<0.00001**
	Cross (fixed, categorical)	Within	0.10 (0.16)		1	12.15	**0.0005**
		Between	0.74 (0.10)				
	F_strain_ (random)			0.22	1	0.00	1
	M_strain_ (random)			0.00	1	0.00	1
	M_strain_:F_strain_ (random)			22.12	1	7.76	**0.0053**
	Residual			273.42			
Female fitness	Block (fixed, categorical)				4	799.11	**<0.00001**
	Cross (fixed, categorical)	Within	0.52 (0.09)		1	2.10	0.1472
		Between	0.67 (0.05)				
	F_strain_ (random)			0.00	1	0.00	1
	M_strain_ (random)			0.00	1	0.00	1
	M_strain_:F_strain_ (random)			5.25	1	6.23	**0.0125**
	Residual			84.89			
Egg-to-adult viability	Block (fixed, categorical)				4	3.38	0.4967
	Cross (fixed, categorical)	Within	0.78 (0.16)		1	44.80	**<0.00001**
		Between	1.65 (0.14)				
	F_strain_ (random)			22.10	1	10.57	**0.0011**
	M_strain_ (random)			3.87	1	0.52	0.4707
	M_strain_:F_strain_ (random)			4.97	1	0.78	0.3758
	Residual			136.20			

## Discussion

Mating status-dependent choice is an elegant theoretical solution to the problem facing polyandrous females of many species—mate rapidly and relatively indiscriminately to avoid virgin death, then choose males with good or compatible genes to maximize reproductive output (Jennions and Petrie 2000). Despite this, there is limited empirical evidence for mating status-dependent choice, and most studies of mate choice tend to focus only on virgin females (Richardson and Zuk 2022). In our experiment (across noncompetitive trials), over 60% of virgin females mated within 20 min, compared with less than 14% of mated females. The observation that virgin females are more responsive to mating is essentially ubiquitous, but it is often interpreted as virgin females being much less choosy. Non-virgin *D. melanogaster* females have been shown to be more discriminatory when presented with males from disparate populations or selection regimens (Kohlmeier et al. 2021; Chechi et al. 2022), but to what extent is this relevant within a population? We observed among-male-strain (M_strain_) variance in virgin latency to mate, strongly suggesting that virgin females are not mating indiscriminately and are indeed exercising ‘choice’, in line with previous evidence (eg (Promislow et al. 1998; Narraway et al. 2010; Katayama et al. 2014)). Critically, we also observed strong correlations between the choices of virgin and non-virgin females within competitive and noncompetitive contexts (though only statistically supported in the latter), indicating that male genotypes that are preferred by non-virgin females are also preferred by virgin females. Thus, in line with a recent meta-analysis (Richardson and Zuk 2022), as well as previous evidence suggesting that *D. melanogaster* females do not “trade-up” (Byrne and Rice 2005), our results do not support mating status-dependent female choice within a natural population of this species, and suggest that virgin females are choosier than anticipated.

One of the key questions asked by Richardson and Zuk (2022) regarding mating status-dependent choice was whether virgin and non-virgin females differ in their avoidance of inbreeding. Here, our viability and fitness data showed clear evidence for inbreeding depression, with flies from within-strain crosses having lower viability and competitive fitness than flies from between-strain crosses. Females thus had the opportunity to avoid the substantial fitness costs associated with inbreeding by preferentially mating with males from different strains. Nevertheless, we found no statistical support for M_strain_-by-F_strain_ interaction variance in mating success, and within-strain mating success was comparable to between-strain mating success, at least within the noncompetitive contexts, demonstrating that females were not avoiding inbreeding. *D. melanogaster* females have previously been shown to favor incestuous matings despite fitness costs (Loyau et al. 2012; Robinson et al. 2012a, 2012b) and, although our results are not suggestive of an inbreeding preference, they provide further evidence that there is no inbreeding avoidance in this species.

Beyond inbreeding avoidance, the presence of M_strain_-by-F_strain_ interaction variance in male and female fitness suggested there was an opportunity for other compatibility-type benefits. Nevertheless, we observed no interaction variance in mating success, suggesting that females from different genetic backgrounds are largely making the same mate choice decisions, a result apparent elsewhere in *D. melanogaster* (Nguyen and Moehring 2019). The lack of support for compatibility-type mating decisions, and the positive genetic correlation of female choices across most contexts, supports the relevance of this species for asking questions about the lek paradox.

We previously demonstrated that sexual selection on male *D. melanogaster* purges deleterious mutations (Dugand et al. 2018, 2019), supporting the genetic benefits of mate choice. Here, we found no statistical support for M_strain_ variance in fitness or viability, precluding a test of the correlation between isofemale strain mating success and fitness. The different experimental design may help to explain these contrasting results. Where we previously used artificial selection in an outbreeding laboratory stock population, here, we used isofemale strains that are maintained as small, inbreeding populations. Consequently, the recessive mutations that led to the evolutionary response in the previous study may be purged in the current design. Likewise, using isofemale strains, Nguyen and Moehring (2019) showed that the sons of successful males had lower productivity, where we have found that successful males have higher productivity in an experiment based on an outbreeding laboratory population (authors, personal communication). When male and female fitness are aligned *versus* when they are antagonistic remains an interesting and important area of study (eg (Long et al. 2012; Singh et al. 2017; Yun et al. 2017; MacPherson et al. 2018)). Of note for *D. melanogaster* may be the effects of body size on purging and sexual conflict. Sexual selection for larger males is common in this species (eg (Partridge and Farquhar 1983; Partridge et al. 1987; Dugand et al. 2018)), but it is not ubiquitous (eg (De Nardo et al. 2021)). Larger males are more harmful to females (Pitnick and García–González 2002; Friberg and Arnqvist 2003), but female choice for larger males can purge mutations (Dugand et al. 2018). Here, males from successful strains tended to be larger, but these strains did not have high fitness or viability. Also, artificial selection for larger size does not purge mutations in the same way that female choice has been shown to (Dugand et al. 2018). Size depends on both genes and the environment, and studies manipulate size through both means. The costs and benefits to females of mating with larger males will, therefore, vary across contexts, and resolving how size impacts various aspects of sexual selection remains an open question.

The presence of M_strain_ variance in male mating success may reflect genetic variance in male latency to court, rather than female choosiness. Male *D. melanogaster* are choosy (Byrne and Rice 2006), and there is genetic variation in their latency to court (Ratterman et al. 2014). However, latency to court is relatively short compared to latency to mate, such that the total time to mate (our measure of mating success) strongly reflects female latency to mate (Ratterman et al. 2014). Given that females ultimately have control over mating (Spieth 1974), our measures of mating success should be representative of female mating decisions. We thus conclude that most of the M_strain_ variance is due to female mating decisions, which are similar whether females have previously mated or not.

Unfortunately, we lacked statistical power to adequately test how multiple males affected mating outcomes across female mating status. For example, correlations were positive and moderately strong, except for that between the choices of non-virgin females in noncompetitive *versus* competitive arenas. In two independent experiments in our laboratory, we have found good genes benefits of sexual selection when non-virgin female *D. melanogaster* chose among multiple males (Dugand et al. 2018; Gibson Vega et al. 2020). In a third experiment, where non-virgin females “chose” through single-male latency to mate trials, we found no such fitness benefits (authors, personal communication). Only when multiple males are present can females choose among potential suitors; arguably, single-male latency trials do not fully capture female choice (Dougherty 2020). Our results may be suggestive of intriguing contrasts across the four contexts, but are ultimately statistically unclear.

Finally, it is worth explicitly outlining that our results do not preclude non-virgin females from being choosier than virgins and, indeed, there is evidence that they are (Kohlmeier et al. 2021; Chechi et al. 2022). Our strongest correlation between the choices of virgin and non-virgin females was 0.8, but compatibility intervals were wide despite not overlapping zero. Thus, although there is strong evidence for alignment, there is ample opportunity for contrasting decisions. Moreover, within our experimental design, non-virgins had up to 7 d respite between their first and second mating, such that their choosiness may have been reduced, and virgin females were over 1 wk old and were reared at a relatively high density, so they may have been relatively choosy. Additionally, flies were collected from a dense population in the wild and were housed in large numbers in the laboratory and, thus, owing to the high encounter rate, virgin females can theoretically afford to be more discriminatory (Jennions and Petrie 2000; Kokko and Mappes 2005).

## Conclusions

The observations that virgin females are more responsive to mating, and that non-virgin female *D. melanogaster* are more discriminate under some circumstances, do not preclude virgin female choice. We show virgin females to be both responsive and choosy. Nevertheless, as Richardson and Zuk (2022) noted, testing the mating decisions of non-virgin females, and the consequences of those decisions, remains an important goal. Our results are indicative of an overall alignment in the choices of virgin and non-virgin females, but they do not preclude important differences in outcomes across contexts. In particular, they hint at the possibility that non-virgin females require multiple males to choose (Dougherty 2020). Our results also add to evidence for both a large-male mating advantage and the absence of inbreeding avoidance, which each support the lek paradox in suggesting directional female mate preferences. That virgin females make similar decisions to previously mated females further strengthens the directional selection on males and enhances this specter of the lek paradox.

## Supplementary Material

araf080_suppl_Supplementary_Figures_Tables_1

## Data Availability

Analyses reported in this article can be reproduced using the data provided by Dugand et al. (2025).
