# Supplementary material for: Mating status-dependent “choice” in competitive and noncompetitive arenas
Source: Behav Ecol. 2025 Aug 28;36(4):araf080. doi: 10.1093/beheco/araf080 (PMC12457708; doi:10.1093/beheco/araf080)
Supplement: araf080_suppl_Supplementary_Figures_Tables_1 [file araf080_suppl_supplementary_figures_tables_1.docx]

**Supplementary Material**

***Supplementary Text***

*Maximum likelihood analysis of non-competitive male mating success*

The following analyses use the same binary data as described in the **Main Text**. Essentially, each male was scored as a winner if they were in the first 25% to mate for a given block and context, and were otherwise scored as a loser.

We first analysed the random effects of M_strain_ (the isofemale strain from which the male was derived), F_strain_ (the isofemale strain from which the female was derived), and their interaction on non-competitive mating success separately for virgin and non-virgin females using binomial generalised linear mixed effects models, implemented in *lme4* (Bates et al. 2015). Cross (within- *versus* between-isofemale strain) was included as a categorical fixed effect. We tested the significance of the interaction term using likelihood ratio tests, where twice the difference in log likelihoods between nested models with and without the interaction term was tested against the χ^2^ distribution with one degree of freedom. In each analysis, we removed this interaction term as it explained no variance (**Table S1**). Note that this interaction term explained zero variance regardless of whether we included the cross fixed effect. We then used likelihood ratio tests to statistically test the presence of M_strain_ and F_strain_ variance against a reduced model that excluded the interaction term: both effects were statistically significant for both contexts (**Table S1**).

To estimate the covariance between contexts, we ran a third model across the full data set (*i.e.*, including all virgin and non-virgin female data). This analysis included M_strain_ and F_strain_ random effects, as well as each of these random effects modelled as interactions with female mating status. Cross, mating status, and their interaction were also included as categorical fixed effects. If, for example, strains with high (low) breeding values for virgin male mating success had high (low) breeding values for non-virgin male mating success, then the M_strain_ variance would still be high, reflecting a positive correlation across contexts. By contrast, if the breeding values varied across the mating status of the female, then most of the variance would be explained by the M_strain_-by-mating status interaction, and the M_strain_ variance would be low. Thus, the covariance matrix for M_strain_, from which we calculated the correlation, has the M_strain_ variances from the single-context analyses (bolded in **Table S1**) on the diagonal, and the M_strain_ variance from the two-context analysis off the diagonal (see pp. 111 (Roff and Wilson 2014)). The covariance for male strain was 0.120, with the correlation, therefore, being 1.067.

Finally, we took a jack-knife approach to estimate the standard error for the correlation, where we performed these three analyses five times, dropping one block each time. We used the five estimated correlations to calculate the standard error as:

$$SE=\sqrt{\left[ \frac{n-1}{n}\sum\left( x_{i}-\bar{x} \right)^{2} \right]}$$

where $x_{i}$ is the *i*th jack-knifed estimate, $\bar{x}$ is the mean from the five estimates, and $n$ is five.

The mean (±2s.e.) correlation was 1.081 (0.769, 1.393), supporting a significant positive correlation between the choices of virgin and non-virgin females.

***Supplementary Figures and Tables***


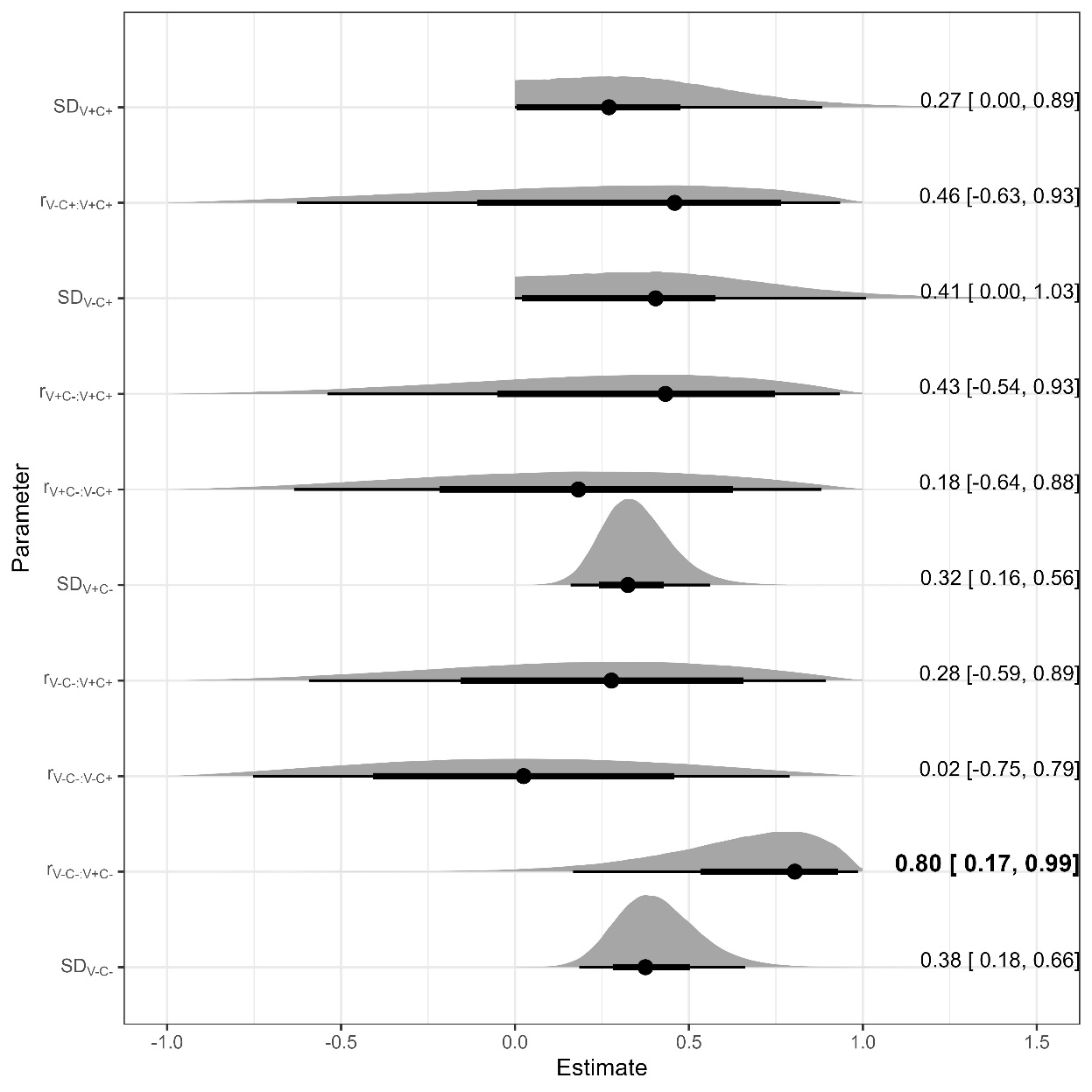


**Figure S1** | Posterior distributions for M_strain_ parameters in the four contexts. Points are posterior modes, and bars are 66% (thick) and 95% (thin) highest density credible intervals. SD is the standard deviation; r is the correlation. Females used in mating trials were virgin (V+) or non-virgin (V-), and were presented to one (C-) or four (C+) males. Bolding is to highlight the correlation where 95% CI do not overlap zero.


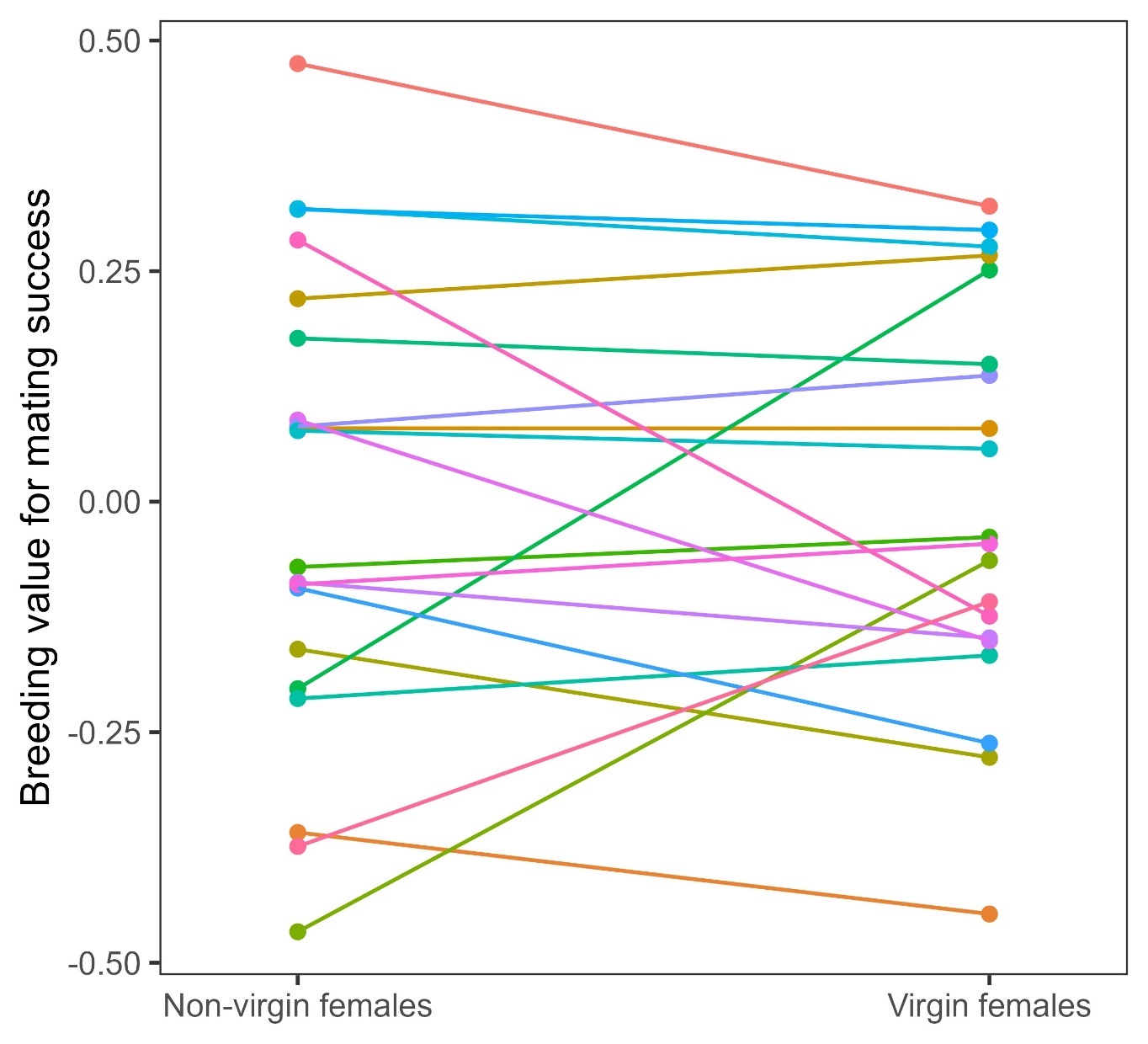


**Figure S2 |** Breeding values for M_strain_ mating success in non-competitive arenas to non-virgin and virgin females (based on the analyses presented in the **Supplementary Text**). Jack-knifing suggested that the correlation was statistically significantly above zero, and not significantly different to one.


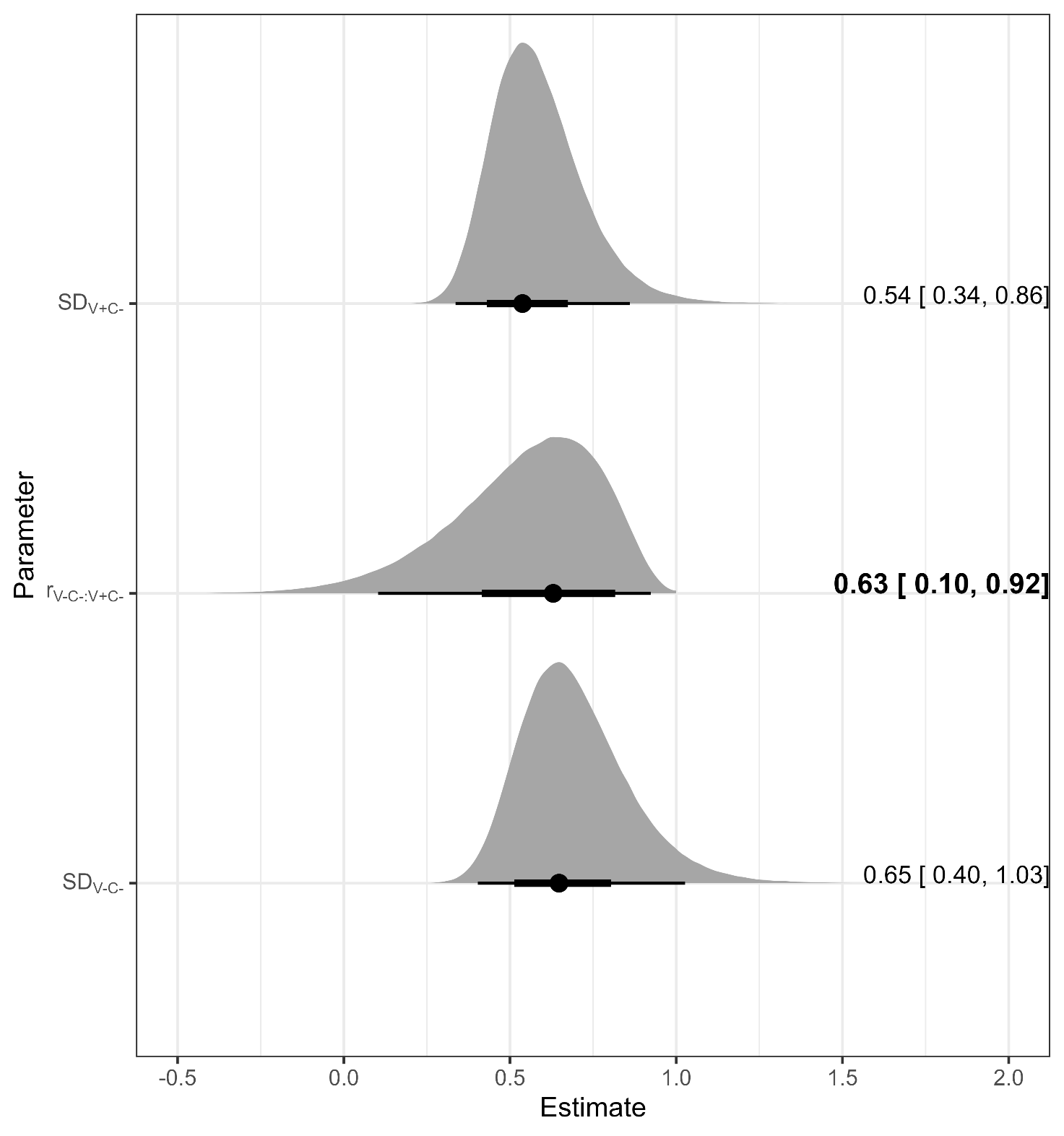


**Figure S3** | Posterior distributions for F_strain_ parameters in the non-competitive contexts (C-). Points are posterior modes, and bars are 66% (thick) and 95% (thin) highest density credible intervals. SD is the standard deviation; r is the correlation. Females used in mating trials were virgin (V+) or non-virgin (V-). Bolding is to highlight the correlation where 95% CI do not overlap zero.

**Table S1** | Mixed effect model results for the non-competitive mating trials. Bolding indicates the values that were used to estimate the M_strain_ correlation across contexts, outlined in the **Supplementary Text**.

| Mating status | Effect | χ^2^ | d.f. | *P* | Variance |
| --- | --- | --- | --- | --- | --- |
| Non-virgin | Cross (fixed, categorical) | 1.21 | 1 | 0.2709 |  |
|  | F_strain_ (random) | 35.44 | 1 | <0.00001 | 0.370 |
|  | M_strain_ (random) | 8.01 | 1 | 0.0047 | **0.132** |
|  | F_strain_:M_strain_ (random) | 0.00 | 1 | 1 | 0.00 |
| Virgin | Cross (fixed, categorical) | 0.69 | 1 | 0.4066 |  |
|  | F_strain_ (random) | 31.98 | 1 | <0.00001 | 0.262 |
|  | M_strain_ (random) | 8.06 | 1 | 0.0045 | **0.096** |
|  | F_strain_:M_strain_ (random) | 0.00 | 1 | 1 | 0.00 |

**Table S2** | Strain means for measured traits. V^-^ and V^+^ are non-virgin and virgin females, respectively, and C^-^ and C^+^ are non-competitive and competitive environments, respectively. WL: wing length (mm); SC: sex comb tooth number (sum across right and left combs); PC1: first PC for wing shape.

| **Block** | **Strain** | **V^-^C^-^** | **V^+^C^-^** | **V^-^C^+^** | **V^+^C^+^** | **WL** | **SC** | **PC1 (x1000)** |
| --- | --- | --- | --- | --- | --- | --- | --- | --- |
| 1 | 3 | 0.40 | 0.35 | 0.30 | 0.35 | 1.31 | 21.07 | 3.06 |
| 1 | 12 | 0.20 | 0.16 | 0.19 | 0.21 | 1.27 | 21.51 | 7.56 |
| 1 | 22 | 0.18 | 0.33 | 0.26 | 0.20 | 1.31 | 21.80 | -7.21 |
| 1 | 42 | 0.22 | 0.16 | 0.25 | 0.24 | 1.26 | 22.74 | -4.97 |
| 2 | 4 | 0.15 | 0.12 | 0.19 | 0.18 | 1.25 | 21.95 | 1.06 |
| 2 | 7 | 0.32 | 0.34 | 0.30 | 0.34 | 1.31 | 22.51 | -13.52 |
| 2 | 32 | 0.18 | 0.20 | 0.32 | 0.24 | 1.25 | 20.53 | 7.71 |
| 2 | 39 | 0.35 | 0.35 | 0.19 | 0.24 | 1.26 | 21.46 | 9.37 |
| 3 | 16 | 0.09 | 0.23 | 0.33 | 0.30 | 1.23 | 21.83 | 11.17 |
| 3 | 21 | 0.19 | 0.24 | 0.33 | 0.25 | 1.20 | 20.43 | -16.33 |
| 3 | 38 | 0.31 | 0.34 | 0.19 | 0.27 | 1.26 | 23.00 | -5.77 |
| 3 | 55 | 0.24 | 0.20 | 0.15 | 0.18 | 1.23 | 23.34 | 11.89 |
| 4 | 6 | 0.27 | 0.28 | 0.33 | 0.28 | 1.23 | 22.51 | 1.52 |
| 4 | 43 | 0.27 | 0.29 | 0.23 | 0.33 | 1.26 | 22.82 | 7.78 |
| 4 | 59 | 0.33 | 0.21 | 0.22 | 0.26 | 1.27 | 21.35 | 15.53 |
| 4 | 60 | 0.13 | 0.23 | 0.22 | 0.13 | 1.28 | 22.51 | -2.55 |
| 5 | 31 | 0.30 | 0.30 | 0.28 | 0.33 | 1.31 | 23.71 | -20.49 |
| 5 | 37 | 0.27 | 0.27 | 0.18 | 0.21 | 1.20 | 24.45 | 1.01 |
| 5 | 51 | 0.22 | 0.20 | 0.09 | 0.07 | 1.28 | 22.55 | -16.39 |
| 5 | 56 | 0.22 | 0.24 | 0.45 | 0.39 | 1.27 | 23.50 | 13.62 |

**Table S3** | Eigenvector loadings for the first principal component of wing landmarks.

| Coordinate | *e_1_* |
| --- | --- |
| x1 | 0.11 |
| y1 | 0.05 |
| x2 | -0.36 |
| y2 | 0.29 |
| x3 | 0.20 |
| y3 | 0.12 |
| x4 | 0.19 |
| y4 | -0.01 |
| x5 | -0.52 |
| y5 | -0.23 |
| x6 | -0.23 |
| y6 | -0.20 |
| x7 | -0.10 |
| y7 | -0.05 |
| x8 | 0.34 |
| y8 | -0.01 |
| x9 | 0.36 |
| y9 | 0.05 |

**Table S4** | Summary of maximum likelihood analyses for morphological traits and egg-to-adult viability. Wing size was power-transformed (y^5^) prior to analysis. Models were (generalised) linear mixed effects models ([g]lmm). CMP is Conway-Maxwell-Poisson distribution. On average, we measured 38.5 males per strain.

| Trait | Model | Effect | Variance(x1000) | d.f. | χ^2^ | *P* |
| --- | --- | --- | --- | --- | --- | --- |
| Wing size (y^5^) | lmm\|Gaussian | Block (fixed, categorical) |  | 4 | 7.90 | 0.095 |
|  |  | Isofemale strain (random) | 56.03 | 1 | 82.89 | <0.00001 |
|  |  | Vial (random) | 3.04 | 1 | 1.54 | 0.214 |
|  |  | Residual | 100.84 |  |  |  |
| Wing shape | lmm\|Gaussian | Block (fixed, categorical) |  | 4 | 1.93 | 0.749 |
|  |  | Isofemale strain (random) | 0.13 | 1 | 164.19 | <0.00001 |
|  |  | Vial (random) | 0.00 | 1 | 0.00 | 1 |
|  |  | Residual | 0.11 |  |  |  |
| Sex comb size | glmm\|CMP | Block (fixed, categorical) |  | 4 | 14.98 | 0.005 |
|  |  | Isofemale strain (random) | 1.08 | 1 | 50.21 | <0.00001 |
|  |  | Vial (random) | 0.02 | 1 | 0.04 | 0.837 |

**References**

Bates, D., M. Mächler, B. Bolker, and S. Walker. 2015. Fitting linear mixed-effects models using lme4. Journal of Statistical Software 67:1-48.

Roff, D. A. and A. J. Wilson. 2014. Quantifying genotype‐by‐environment interactions in laboratory systems. Pp. 100-136 *in* J. Hunt, and D. J. Hosken, eds. Genotype‐by‐environment interactions and sexual selection. John Wiley & Sons, Ltd, Chichester.
